# Who gets included? A scoping review protocol of digital health interventions for older adults with heart failure through an equity lens

**DOI:** 10.1371/journal.pone.0337990

**Published:** 2025-12-04

**Authors:** Kristina M. Kokorelias, Peter Hoang, Maurita T. Harris

**Affiliations:** 1 Division of Geriatric Medicine, Department of Medicine, Sinai Health System and University Health Network, Toronto, Ontario, Canada; 2 Department of Occupational Science & Occupational Therapy, Temerty Faculty of Medicine, University of Toronto, Toronto, Ontario, Canada; 3 National Institute on Ageing, Toronto Metropolitan University, Toronto, Ontario, Canada; 4 Rehabilitation Sciences Institute, Temerty Faculty of Medicine, University of Toronto, Toronto, Ontario, Canada; 5 Institute of Health Policy, Management and Evaluation, University of Toronto, Toronto, Ontario, Canada; 6 Faculty of Liberal Arts, Wilfrid Laurier University, Waterloo, Ontario, Canada; Mayo Clinic College of Medicine and Science, UNITED STATES OF AMERICA

## Abstract

**Introduction:**

Heart failure is a common and progressive condition that significantly impacts older adults, leading to increased morbidity, reduced quality of life, and healthcare utilization. As its prevalence continues to rise, there is a need for effective management strategies tailored to this population. Digital health interventions (DHIs) have emerged as promising tools for managing chronic conditions like heart failure, potentially improving accessibility and personalizing care. However, there is limited understanding of the inclusivity and effectiveness of these interventions across diverse subgroups of older adults, particularly those differentiated by age, cognitive status, socioeconomic status, sex/gender, and race/ethnicity.

**Methods:**

This protocol outlines a scoping review to assess the extent of literature on DHIs for managing heart failure among older adults, focusing on the representation of diverse subgroups and the characteristics of the interventions. Specifically, the review will explore which populations are included in current DHIs, how they are represented, and how intervention characteristics influence participation and outcomes. This scoping review will follow the Joanna Briggs Institute methodology for scoping reviews, using the PROGRESS-Plus framework to assess equity-related factors such as socioeconomic status, race/ethnicity, geographic location, and cognitive status. The review will focus on randomized controlled trials published between January 1, 2005, and the present, in high-income countries.

**Conclusions:**

The forthcoming scoping review will provide a comprehensive mapping of the existing literature on digital health interventions for heart failure management in older adults, focusing on the inclusivity of diverse subgroups. By identifying gaps in the representation of key demographic factors such as age, cognitive status, socioeconomic status, sex/gender, and race/ethnicity, the review will highlight areas for future research and inform the development of more equitable, effective digital health solutions for heart failure. The findings will be valuable for healthcare practitioners, policymakers, and researchers seeking to improve the accessibility and impact of DHIs in managing heart failure among older populations.

## Introduction

Older adults represent a unique and growing population of heart failure patients who often experience worse clinical outcomes due to age-related physiological changes and comorbidities, such as hypertension, diabetes, and chronic kidney disease [1,2]. These comorbidities, combined with age-related declines in cognitive and functional capacities, complicate the management of heart failure and increase the risk of hospitalization and mortality [2]. Moreover, older adults often face barriers to accessing healthcare services, including transportation limitations, physical frailty, and social isolation, making traditional healthcare models less effective for this group [3,4].

Management of heart failure typically involves a combination of pharmacological treatment, lifestyle modifications, and regular monitoring of clinical symptoms [5,6]. However, due to the chronic and fluctuating nature of the condition, traditional care models often struggle to meet the needs of older adults with heart failure, who face unique challenges related to comorbidities, polypharmacy, cognitive decline, and limited mobility [3,7]. Consequently, there is growing interest in leveraging digital health interventions to address some of these challenges and provide more personalized, accessible, and efficient care [8].

Recently, digital health interventions, including telemedicine, mobile health applications, and remote monitoring devices, have emerged as promising tools for managing chronic conditions, including heart failure [8,9]. The potential for digital health interventions (DHIs) to improve chronic disease management, particularly in populations with complex health needs like older adults with heart failure, is substantial [8,9]. These technologies have the potential to address care gaps by increasing access to care, supporting early identification of decompensation, and promoting more equitable models of home-based and continuous care [10–12]. However, despite the growing implementation of DHIs in cardiovascular care, questions remain about *who* is actually included in these interventions and how effective they are across diverse subgroups of older adults.

Existing systematic reviews of digital health interventions in heart failure have typically focused on broad populations and have treated older adults as a single, homogenous group, without accounting for the diversity within this demographic [13,14]. One recent systematic review examined the effectiveness of digital health interventions in heart failure, specifically in rural U.S. populations [15]. This review provided useful insights into the feasibility and outcomes of digital interventions in underserved settings, including improved clinical management and reduced hospitalization rates [15]. However, its focus was geographically and contextually narrow, excluding broader demographic diversity and international applicability. It also did not explicitly examine how older adults are represented as a distinct subgroup, nor did it consider key equity-related factors, such as differences in age (e.g., younger-old vs. older-old), cognitive status (including normal age-related cognitive changes, mild cognitive impairment, or dementia), socioeconomic conditions, sex and gender, or racial and ethnic identity, that can influence access to and effectiveness of digital health interventions. This is problematic because while digital health interventions, in general, are often promoted as scalable solutions for improving care access and outcomes [16], they may inadvertently reinforce or widen existing inequities if they are not accessible, acceptable, or effective for certain groups—particularly older adults who may experience age-related barriers to technology use or who are marginalized due to factors such as poverty, gender, racialization, geographic isolation, or language [17,18]. Without this information, we risk continuing to design and implement interventions that do not reflect the realities of those most affected by heart failure and most in need of support. Understanding what digital health interventions exist and who they serve is essential to informing equity-oriented digital health policy and practice.

Given the complexity and diversity of the interventions and target population, a scoping review is the most appropriate methodology for this work. While systematic reviews are often used to assess the effectiveness and compare outcomes quantitatively, scoping reviews are more suitable for exploring broad questions, mapping the nature and extent of evidence, identifying knowledge gaps, and clarifying how concepts are used and populations represented [19]. A preliminary search of MEDLINE, the Cochrane Database of Systematic Reviews and *JBI Evidence Synthesis* was conducted and no current or underway systematic reviews or scoping reviews on the topic were identified.

The objective of the forthcoming scoping review is to assess the extent of the literature on digital health interventions for managing heart failure among older adults, specifically evaluating the representation of diverse subgroups. The review aims to identify the characteristics of the interventions and explore how, if at all, they engage diverse participants.

In this review, we conceptualize equity according to the World Health Organization definition: the absence of unfair, avoidable differences in health among groups. While demographic (e.g., age, sex), clinical (e.g., comorbidities, cognitive status), and socioeconomic (e.g., income, housing) factors may influence health outcomes, they do not themselves constitute inequity [20]. Rather, inequities arise when these factors intersect with structural barriers, such as systemic racism, sexism, differential digital literacy, or limited access to technology [20]. Accordingly, we focus on how digital health interventions for heart failure perform across populations with varying levels of access, usability, education, training requirements, connectivity, and cost. This approach allows us to identify potential inequities in intervention use and benefit without conflating demographic or clinical characteristics with inequity itself.

### Review question

1. Who are the actual participants represented in existing digital health interventions aimed at supporting heart failure management in older adults?

Sub-Questions:

1. How are older adults represented in digital health interventions for heart failure, including subgroups differentiated by:
  Age: younger-old (e.g., 60–74 years) versus older-old (e.g., 75+ years)
  Cognitive status: baseline cognitive functioning, including normal age-related cognitive changes, mild cognitive impairment, or dementia
  Socioeconomic background: income, education, housing stability, and employment status
  Sex and gender: self-reported sex and/or gender identity
  Racial and ethnic identity: as reported in the study
  Geographic location: urban vs. rural, or region-specific factors that may affect access or use
2. What are the features of the digital health interventions, including
  Type of technology: mobile apps, telemonitoring devices, web platforms, or wearable sensors
  Delivery method: synchronous (e.g., live video) versus asynchronous (e.g., automated messaging)
  Frequency and duration of use: how often and for how long participants interact with the intervention
  Other features: data sharing capabilities, usability requirements, training or support needed, connectivity requirements, and cost considerations

## Methodology

This scoping review will follow the methodology outlined by the Joanna Briggs Institute (JBI) for scoping reviews, which provides a systematic approach to mapping the breadth of existing literature on a given topic [21]. Our review will be conceptually informed by the Progress Plus framework, which emphasizes the need to examine the effectiveness and equity of interventions, focusing on the representation of diverse populations and their specific needs [22]. PROGRESS-plus identifies the factors that differentiate health opportunities and outcomes and that can contribute to inequalities [22]. Furthermore, the protocol for this review has been designed according to the Preferred Reporting Items for Systematic Reviews and Meta-Analyses Protocols (PRISMA-P) guidelines [23], and the results will be reported in line with the PRISMA Scoping Review (PRISMA-Scr) checklist. The protocol was also registered on Open Science Framework (osf.io/2a7hz).

### Research team

The authors recognize that our perspectives shape the interpretation of equity in digital health interventions. The research team brings expertise in geriatrics, caregiving, and implementation science. One author contributes clinical experience as a geriatrician. Our combined backgrounds inform our focus on older adults, health equity, and methodological rigor. We are attentive to potential biases introduced by our professional backgrounds by using structured and transparent methods, including standardized and piloted data extraction forms, independent dual screening and extraction, and discussion of discrepancies among reviewers. Additionally, the use of the PROGRESS-Plus framework ensures that equity considerations are systematically applied across all included studies, helping to mitigate subjective interpretation.

### Inclusion criteria

#### Participants.

This scoping review will include studies that focus on older adults (aged 50 years and older) diagnosed with heart failure. Although the conventional threshold for older adulthood is 65 years, the decision to include individuals starting at age 50 is informed by health equity considerations and evidence of accelerated aging in certain marginalized populations [24]. Several groups, including individuals experiencing homelessness [25], those living with HIV [26], Indigenous populations [27], and racialized and socioeconomically disadvantaged communities [28], often experience premature onset of age-related comorbidities, including cardiovascular disease and heart failure. Participants may be community-dwelling or reside in institutional or supportive care environments, provided they are involved in an intervention specifically targeting heart failure management.

#### Concept.

The core concept of this review is digital health technology, defined as the use of digital platforms or tools that leverage computing and telecommunications to deliver, enhance, or monitor healthcare services [29]. For the purpose of this review, *digital health interventions* refer to technologies that support the delivery, management, or self-management of heart failure using digital platforms. This includes patient-facing tools that enable communication, monitoring, or data exchange between patients and healthcare providers. Digital health interventions may also include, but are not limited to, mobile health (mHealth) applications, wearable devices, web-based platforms, remote monitoring systems, telemedicine integrated with patient portals, and other digital tools that provide data-driven, interactive, and personalized care for heart failure management [29]. These technologies are typically used to support symptom tracking, medication adherence, self-care education, virtual consultations, or real-time clinical monitoring [30]. Examples include:

- Telemonitoring platforms or mobile applications that allow patients to report symptoms or biometric data (e.g., blood pressure, weight) for clinician review
- Self-management or adherence tools, such as smartphone apps or web-based programs designed to support medication adherence, physical activity, or lifestyle changes
- Remote consultation or communication tools, such as video visits or secure messaging systems

Interventions that are solely provider-facing (e.g., clinical decision support systems without patient interaction) or that do not use a digital interface are excluded.

#### Context.

The context for this review is limited to high-income countries, as classified by the World Bank [31]. We acknowledge that limiting to HICs does not guarantee uniformity in healthcare infrastructure, digital maturity, broadband access, or technology availability. However, this criterion provides a manageable scope for the review and focuses on settings where digital health interventions are more likely to be implemented, studied, and reported in the literature. Included studies may take place in diverse healthcare environments, such as outpatient clinics, home settings, hospital transitional care, rehabilitation centers, or community-based programs, provided they report on outcomes of digital health interventions for older adults with heart failure. Studies conducted in low- or middle-income countries are excluded to avoid additional heterogeneity introduced by substantially different healthcare systems and digital infrastructure.

In addition to capturing information about intervention design and outcomes, this review will pay particular attention to how equity-related factors are reported, using the PROGRESS-Plus framework to assess whether and how participant characteristics such as place of residence, race/ethnicity, socioeconomic status, education level, gender, or other relevant variables are considered. This approach is central to understanding the breadth of digital health interventions available for older adults with heart failure and the inclusivity and applicability of these interventions across diverse populations.

#### Types of sources.

Only randomized controlled trials (RCTs) will be included in this scoping review to manage the scope of the literature and allow for the synthesis of interventions that have undergone rigorous evaluation [32]. RCTs also often provide standardized reporting of participant characteristics, making them well-suited for examining how equity-related variables, such as age, sex/gender, race/ethnicity, socioeconomic status, and cognitive status, are represented and considered in digital health interventions [33]. While we acknowledge that observational and qualitative studies may offer insights into user experiences and contextual factors, these study designs are outside the scope of this review, which aims to synthesize evidence on interventions with established efficacy. We will include peer-reviewed publications written in English or that can be translated into English using Google Translate [34]. Studies that report protocols, observational research, quasi-experimental designs, or qualitative-only findings will be excluded. Conference abstracts and theses will also be excluded.

#### Search strategy.

A three-step search strategy will be utilized in accordance with the JBI methodology for scoping reviews. First, an initial limited search of MEDLINE (via PubMed) and CINAHL (via EBSCOhost) was conducted to identify relevant articles related to digital health interventions in the management of heart failure among older adults. The keywords and index terms used in the titles and abstracts of identified articles were analyzed to inform the development of a comprehensive search strategy. This strategy was iteratively refined and adapted for use across all selected databases.

In the second step, a full electronic search strategy will be implemented across the following databases: MEDLINE (PubMed), CINAHL (EBSCOhost), Embase (Elsevier), and Scopus (Elsevier). The search strategy will include a combination of controlled vocabulary terms (e.g., MeSH, Emtree) and free-text terms related to digital health interventions, heart failure, and older adults. Filters for randomized controlled trials will be applied where appropriate. The final search strategies for each database, including all identified keywords and index terms, will be presented in the final publication. A sample of keywords is presented in Table 1. The strategy will be reviewed by a medical librarian with expertise in systematic searching to ensure completeness and accuracy.

**Table 1:** Keywords.

Keyword	Synonyms
Heart Failure	Heart Failure, Diastolic; Cardio-Renal Syndrome
Cardiovascular diseases	Cardiovascular abnormalities; Cardiovascular infections; Heart diseases;
Digital Health Technologies	eHealth; mHealth; Telehealth; Health Informatics; Remote Monitoring; clinical decision support tools

The third step will involve hand-searching the reference lists of all included studies to identify any additional relevant publications. References from systematic reviews on similar topics will also be screened to identify studies not captured in the database searches. No automated tools will be used to identify additional articles beyond database and reference list screening.

Only peer-reviewed primary studies published between January 1, 2005, and the present will be included. This 20-year time frame reflects the accelerated growth and relevance of digital health technologies in clinical practice, particularly following major mobile, internet-based, and wearable developments. Earlier studies are unlikely to reflect current digital infrastructure, patient usage patterns, or intervention designs and were therefore excluded.

All retrieved records will be uploaded to a reference management software (i.e., Covidence for duplicate removal and screening [35].

#### Study/Source of evidence selection.

Before full screening begins, a pilot screening process will be conducted to ensure consistent application of the inclusion and exclusion criteria. The review team will screen a random sample of at least 25 records independently. This pilot will be repeated iteratively until inter-rater agreement reaches a Cohen’s kappa statistic of >0.70, indicating sufficient reliability among screeners [36]. During the pilot phase, discrepancies will be discussed, and the screening criteria may be refined for clarity and consistency. Once this threshold is achieved, the remaining citations will be screened.

Following the pilot, two reviewers will independently perform title and abstract screening. Any citations deemed potentially eligible or unclear will proceed to full-text review. Full-text articles will also be screened independently by two reviewers. Disagreements at any stage will be resolved through discussion or by consulting a third reviewer if consensus cannot be reached.

Reasons for exclusion of full-text articles that do not meet the inclusion criteria will be documented. The study selection process will be summarized in a PRISMA-ScR flow diagram, detailing the number of studies screened, assessed, included, and excluded, along with justifications for exclusions [37].

#### Data extraction.

Data will be extracted from papers included in the scoping review by two independent reviewers using a data extraction tool on Covidence. Two reviewers will extract data independently using a standardized and piloted data extraction form (See Sample in Supplemental Material 2). The form will be developed based on JBI methodology and adapted to capture equity considerations through the PROGRESS-Plus framework, which includes: Place of residence, Race/ethnicity/culture/language, Occupation, Gender/sex, Religion, Education, Socioeconomic status, and Social capital, as well as additional factors such as age, disability, sexual orientation, and health literacy [22]. Where available, we will also extract and report participants’ working or employment status to examine how engagement with digital health interventions may vary between those who are still employed and those who are retired.

The data extraction form will be piloted on a small sample of included studies (n=~2) to ensure clarity and consistency. Revisions will be made based on team consensus, and inter-rater reliability will be monitored. Discrepancies will be resolved through discussion or by a third reviewer when necessary.

We will extract the following information:

- Study Characteristics: Author(s), year of publication, country, study setting (e.g., rural/urban), funding source, study design, recruitment techniques
- Participant Characteristics: Age (including age ranges and means), sex/gender, racial/ethnic background (if reported), socioeconomic indicators (e.g., income, housing status, employment), education level, digital literacy, language proficiency, comorbidities, and indicators related to other PROGRESS-Plus factors (e.g., cognitive status, geographic isolation, disability)
- Intervention Characteristics:
  Type of digital health intervention (excluding phone-only interventions)
  Technology/platform used (e.g., apps, remote monitoring devices, web portals, wearables)
  Targeted functions (e.g., symptom monitoring, medication adherence, health coaching)
  Mode of delivery and duration
  Involvement of healthcare providers
  Co-design with patients or marginalized communities (if applicable)
  Efficacy of the intervention (yes/no; based on statistically significant outcomes if reported)

• Equity Considerations:
  ◦ Whether the intervention was designed or adapted for specific subgroups (e.g., rural populations, those with limited digital literacy)
  ◦ Strategies to enhance digital inclusion (e.g., technical support, training, accessibility adaptations)
  ◦ Explicit reporting of subgroup outcomes or disparities
• Limitations and Implications:
  ◦ Authors’ discussion of equity limitations or gaps
  ◦ Recommendations for future research with equity implications

Where possible, extracted data will be disaggregated by equity-relevant variables. If data are missing or unclear, we will attempt to contact the study authors for clarification. Extracted data will be stored securely on institutional servers, and, where possible, de-identified extraction tables will be made available in an open-access repository upon publication.

#### Data analysis and presentation.

Data will be organized and summarized using both quantitative and qualitative descriptive approaches. Quantitative data (e.g., publication year, study setting, sample characteristics, intervention type) will be analyzed using descriptive statistics, such as frequencies and proportions, to provide a numerical overview of the included studies [38]. This synthesis will be guided by deductive coding informed by the PROGRESS-Plus framework, which includes the following equity domains: Place of residence, Race/ethnicity/culture/language, Occupation, Gender/sex, Religion, Education, Socioeconomic status, and Social capital, along with additional factors such as age, disability, sexual orientation, and health literacy [22]. Equity-related data will be synthesized using a structured analytic approach informed by the PROGRESS-Plus framework. We will quantify the frequency of reporting for each equity factor (e.g., age subgroups, sex/gender, race/ethnicity, socioeconomic indicators, cognitive status) and identify missing variables across studies. Where possible, we will cross-tabulate equity factors with intervention characteristics, such as technology type, mode of delivery, accessibility strategies, and co-design elements. We will also conduct a thematic analysis of reporting patterns to assess whether subgroup inclusion is superficial or substantive. This approach ensures that equity considerations are analyzed systematically and meaningfully, beyond descriptive reporting alone. By linking equity variables to intervention characteristics, we aim to identify features such as co-design, training, or accessibility strategies that may enhance inclusion and engagement of underrepresented subgroups in digital health interventions for older adults with heart failure.

The synthesis will highlight patterns in representation, reporting, and outcomes related to equity-relevant subgroups, identify gaps in the literature, and provide insight into how digital health interventions may reinforce or mitigate disparities among older adults with heart failure.

As a scoping review, no formal statistical hypothesis testing will be performed. Extracted data will be summarized using descriptive quantitative measures (e.g., frequencies, proportions) and qualitative narrative synthesis to highlight patterns, gaps, and equity considerations across studies.

All co-authors will contribute to study design, screening, data extraction, synthesis, and manuscript preparation. All authors will participate in interpreting findings and drafting the manuscript.

## Discussion

This forthcoming scoping review aims to map and critically analyze the landscape of digital health interventions for heart failure management among older adults, with a specific focus on how diverse subgroups are represented and supported. The need for this review is driven by the growing prevalence of heart failure in aging populations, coupled with rising interest in using digital health tools to manage chronic conditions in community and home settings. While digital health interventions are often framed as scalable solutions to address gaps in care delivery, the extent to which these technologies are equitably designed, implemented, and evaluated for older adults—particularly those with intersecting vulnerabilities—remains largely unclear. Despite the promise of digital health, older adults often face significant barriers to adoption and sustained engagement. These include digital literacy challenges, sensory or cognitive impairments, lack of access to reliable internet or devices, and mistrust or discomfort with technology. If interventions are not designed with these barriers in mind, they risk precisely excluding the individuals who might benefit most. Moreover, many digital health interventions rely on assumptions about patients’ social support systems, caregiving structures, and home environments, which may not hold true for isolated or marginalized older adults. This review will help identify whether such contextual factors are considered in intervention design and evaluation, or if they are overlooked.

The use of the PROGRESS-Plus framework in this review is a deliberate effort to move beyond surface-level descriptions of study populations and interrogate how (and whether) digital health interventions reflect the real-world diversity of older adults living with heart failure. This includes examining characteristics such as socioeconomic status, gender, race/ethnicity, cognitive function, and rural or remote residence. By explicitly assessing how these variables are reported and addressed in randomized controlled trials, the review will contribute to a more equity-oriented understanding of who benefits from these technologies and under what conditions.

### Limitations

This scoping review has several limitations that should be acknowledged. First, by including only RCTs, we may exclude valuable insights from observational studies, implementation studies, and qualitative research that explore contextual factors, user experiences, and barriers to digital health adoption for older adults living with heart failure. Second, the review is restricted to studies conducted in high-income countries. This criterion was chosen to ensure consistency in health system infrastructure and digital health readiness; however, it limits the generalizability of findings to lower-resource settings or middle-income countries where heart failure is also prevalent and where different barriers to digital health use may exist. Although this review focuses on high-income countries to provide a feasible scope, we recognize that digital divides exist within these settings and that findings may not fully generalize to low- and middle-income contexts. Future research should explore equity considerations in digital health interventions in diverse resource settings to provide a more global perspective. Third, we restricted our inclusion criteria to studies published in English or translatable to English via Google Translate. As a result, potentially relevant evidence published in other languages may have been inadvertently excluded, particularly from non-English-speaking high-income countries. Fourth, this review focuses exclusively on studies that target older adults aged 50 and above with a formal diagnosis of heart failure. While this decision reflects an equity-oriented approach that considers accelerated aging in certain populations, it may exclude interventions designed for general populations or younger adults that could still be relevant to the needs of older adults. Fifth, the scope of our review does not include grey literature such as unpublished studies, dissertations, or reports from health agencies or technology developers. As a result, potentially informative real-world implementation data or equity-focused evaluations may not be captured.

We also acknowledge that few studies are powered to assess outcomes across subgroups, and that many digital health interventions evolve after initial publications. Therefore, this review focuses on describing the characteristics of study populations and intervention features as reported in the literature, rather than making broad generalizations about equity in digital health interventions. This approach provides a mapping of representation and design considerations over the past 20 years, while recognizing that later iterations of interventions may differ from what is published.

While this scoping review focuses on RCTs to assess intervention efficacy, we recognize that qualitative and mixed-methods studies can provide important equity-relevant insights regarding usability, cultural appropriateness, and implementation barriers. Future work may include a complementary qualitative synthesis to address these contextual factors and provide a more complete understanding of equity in digital health interventions for older adults with heart failure.

## Conclusion

The forthcoming scoping review highlights the growing body of evidence on digital health interventions for managing heart failure in older adults, with an emphasis on equity. While promising advancements have been made in the development and implementation of these interventions, several gaps remain in understanding their equitable access, uptake, and impact across diverse older adult populations. There is a clear need for more research that not only examines the clinical efficacy of digital health interventions but also assesses their real-world applicability, usability, and equity outcomes. By synthesizing the current evidence, this review underscores the importance of integrating equity considerations into the design, implementation, and evaluation of digital health interventions for heart failure care. As digital health continues to evolve, it is crucial that healthcare systems ensure that these innovations are accessible, effective, and tailored to the unique needs of all older adults living with heart failure.
